# Seasonal Variation in Stable Carbon and Nitrogen Isotope Values of Bats Reflect Environmental Baselines

**DOI:** 10.1371/journal.pone.0117052

**Published:** 2015-02-20

**Authors:** Ana G. Popa-Lisseanu, Stephanie Kramer-Schadt, Juan Quetglas, Antonio Delgado-Huertas, Detlev H. Kelm, Carlos Ibáñez

**Affiliations:** 1 Estación Biológica de Doñana, Consejo Superior de Investigaciones Científicas (CSIC), Sevilla, Spain; 2 Estación Experimental del Zaidín, Consejo Superior de Investigaciones Científicas (CSIC), Granada, Spain; 3 Leibniz Institute for Zoo and Wildlife Research (IZW), Berlin, Germany; University of Regina, CANADA

## Abstract

The stable carbon and nitrogen isotope composition of animal tissues is commonly used to trace wildlife diets and analyze food chains. Changes in an animal’s isotopic values over time are generally assumed to indicate diet shifts or, less frequently, physiological changes. Although plant isotopic values are known to correlate with climatic seasonality, only a few studies restricted to aquatic environments have investigated whether temporal isotopic varia-tion in consumers may also reflect environmental baselines through trophic propagation. We modeled the monthly variation in carbon and nitrogen isotope values in whole blood of four insectivorous bat species occupying different foraging niches in southern Spain. We found a common pattern of isotopic variation independent of feeding habits, with an overall change as large as or larger than one trophic step. Physiological changes related to reproduction or to fat deposition prior to hibernation had no effect on isotopic variation, but juvenile bats had higher δ^13^C and δ^15^N values than adults. Aridity was the factor that best explained isotopic variation: bat blood became enriched in both ^13^C and ^15^N after hotter and/or drier periods. Our study is the first to show that consumers in terrestrial ecosystems reflect seasonal environmental dynamics in their isotope values. We highlight the danger of misinterpreting stable isotope data when not accounting for seasonal isotopic baselines in food web studies. Understanding how environmental seasonality is inte-grated in animals’ isotope values will be crucial for developing reliable methods to use stable isotopes as dietary tracers.

## Introduction

Stable isotope analysis is considered a powerful tool to study wildlife diets. The isotopic composition of an animal’s body closely reflects the isotopic composition of the diet, plus a predictable isotopic enrichment [1, 2] called discrimination factor. The isotopic value of an animal’s tissue may change over time. The most straightforward cause is a switch to a new diet that is isotopically distinct from the previous diet [3–6], or a change in the proportional contributions of dietary sources consumed, each source with a distinct isotopic signature. These proportions are typically calculated with the help of mixing models that use as parameters the animal’s isotope values before and after the presumed change, the isotopic signatures of the potential diet sources, which are assumed to be in temporal equilibrium, and a fixed estimate of the diet-tissue discrimination factors [7, 8].

Even in the absence of dietary variation, an animal’s tissues may still undergo significant isotopic variation of a dietary origin. This could be a result of the organisms that constitute the animal’s diet not being in temporal isotopic equilibrium, whether because of direct dietary changes of these organisms, or because of isotopic variation at lower trophic levels [9] that propagates up the food chain.

Plant isotopic values have indeed been shown to fluctuate in response to a number of environmental factors, often following a seasonal pattern [10–16]. These factors affect stomatal aperture and conductance in the leaf and consequently the stable carbon discrimination between atmospheric carbon dioxide and the leaf’s fixed carbon [11]. Carbon isotope values of leaves typically increase with drought both on a geographical [12, 16, 17] and a temporal scale [18–20]. Similarly, nitrogen isotope values of plant material are typically negatively correlated with precipitation across geographical gradients [21, 22]. Recent studies also report a relationship, albeit less consistent in direction, between temporal variability in plant nitrogen isotope values and climatic seasonality [23–25].

Temporal isotopic changes in consumers could thus be tracked back to seasonal environmental baselines that affect producer isotopic signals. However, dietary reconstructions based on stable isotopes do not generally take into account this source of variation, and only few studies, conducted in aquatic environments [8, 26, 27], have explored the relationship between temporally changing environmental conditions and isotopic changes in consumers’ tissues. In fact, recent studies in freshwater and marine ecosystems warn against the common practice of using stable isotopes in food web studies, both in aquatic and terrestrial systems, without first investigating and accounting for dynamic baselines [8, 28, 29]. While environmental baselines may not be an issue when analyzing a tissue with a time of integration long enough to even out these patterns, or when combining different tissues with varying turnover rates to account for temporal change, they may introduce bias in many other situations, and should in any case be investigated before disregarding their significance.

In addition to diet, variations in isotope values of animal tissues may also result from a change in physiological conditions that affect the discrimination process between diet and consumer [9]. Reproduction [30, 31], growth [32, 33], nutritional stress and starvation [33–35], and water stress [36] have been suggested to alter an animal’s isotopic composition, yet their effect, if any, is usually considered small compared to the direct effect of diet.

Popa-Lisseanu et al. [37] used stable isotope analysis to verify a switch from insectivory to carnivory in the giant noctule bat, *Nyctalus lasiopterus*, during periods of bird migration in autumn and spring, when bats preyed on migratory birds [38]. The seasonal change in bat blood isotope values from periods without bird migration to migratory periods matched the difference between insect and bird isotope values. In addition, bat blood isotope values correlated strongly with the annual pattern of density of birds on migration and proportional amount of feathers found in bat feces. While these results pinned down the diet switch from an insect to a bird diet as the most likely cause of the seasonal isotopic changes observed, other potential causes, such as physiological condition or environmental baselines could not be ruled out.

To improve understanding of the factors driving temporal isotopic variation of higher-level consumers in terrestrial ecosystems, we explored seasonal fluctuations of stable carbon and nitrogen isotope values in blood of bats from Andalusia, Spain. Using a general linear model, we investigated the effect of bat physiology (reproduction, hibernation, or factors related to age), climatic variation, and species (each studied species occupying a specific foraging niche) on temporal dynamics of bat blood isotope values.

## Materials and Methods

### Study area and study species

We conducted the study in West Andalusia (southwestern Spain) in the provinces of Seville and Cádiz. Climate is Mediterranean and highly seasonal. Winters are mild with a mean ambient temperature of 10°C in January, and summers are hot with a mean ambient temperature of 27°C in July and August. Mean annual rainfall is about 550 mm, November and December being the months with the highest precipitation, and the period June-August with the lowest and close to 0 [39].

We collected blood samples from three strictly insectivorous bat species with different feeding habits: 1) the medium-large aerial-hawker *Eptesicus isabellinus* (body mass (bm) = 22 g) which feeds on hard-bodied flying insects, mainly Coleoptera and Hemiptera [40]; 2) the medium-small aerial-hawker *Miniopterus schreibersii* (bm = 12g), which hunts small- to medium-sized winged insects, mostly Lepidoptera but also Diptera and other seasonally abundant insects [41, 42]; and 3) the surface-gleaning *Myotis myotis* (bm = 24g), which feeds on ground arthropods such as carabid beetles, orthopterans and lepidopteran larvae [43, 44]. Additionally, we used own published data on the large aerial-hawking bat *Nyctalus lasiopterus* (bm = 50g), which preys opportunistically on a high variety of large winged insects [45, 46] and seasonally on nocturnally migrating birds [37, 38, 47].

*Eptesicus isabellinus* were captured from a breeding colony in Alcalá del Río, Sevilla (37°31'N, 5°58'W), a town located on the western margin of the Guadalquivir River and surrounded by agricultural land (mainly irrigated crops including cotton, corn and orange trees; [48]). Adult females and juveniles of both sexes roost in the wall crevices of a hydroelectric dam from spring to autumn, when they disperse to unknown wintering roosts.

*Myotis myotis* and *Miniopterus schreibersii* were captured in all-year, mixed-sex colonies in a natural pit cave in Villamartín, Cádiz (36°48'N, 5°35'W). The cave is located on a hillside at the interface between agricultural land (irrigated and non-irrigated cereal and sunflower crops) and natural vegetation of the Cádiz mountain system (Mediterranean shrubs and cork oaks). It hosts a high bat species diversity (*Myotis myotis, M. blythii, M. escalerai, Miniopterus schreibersii, Rhinolophus euryale, R. hipposideros* and *R. ferrumequinum*) and high bat numbers during the breeding season (up to 3000 individuals). Cave temperature remains ca. 20–22ºC year-round. For this reason, the ca. 100–300 individuals (several species) that spend the winter in the cave do not hibernate and emerge to forage. Some females of *M. myotis* even reproduce during winter, outside the normal breeding period for temperate-zone bats in the Northern Hemisphere (from May to July). This is a very rare phenomenon in temperate bats which has hitherto only been reported once for *Myotis myotis* in Spain, in a roost of similar microclimatic conditions [49].

Published data on *Nyctalus lasiopterus* used in this study [37] were obtained during 2002–2004 from breeding colonies (almost exclusively adult females and juveniles of both sexes) in urban parks of Seville (37°22'N, 5°59'W) and Jerez de la Frontera, Cádiz (36°41'N 6°08'W) and in Doñana National Park, Huelva (36°59'N 6°26'W). The bats roosted either in natural tree cavities (Seville), in palm trees of the genus *Washingtonia*, in the space between the old, dried fronds and the trunk (Seville and Jerez), or in bat boxes placed on tree trunks (Doñana).

### Capture and sampling

Bats were captured by placing mist-nets in front of their roosts at dusk. Capture and sampling took place at monthly intervals (on day 15 ± 2 of each month) between August 2004 and October 2005. Ca. 15 individuals of each species were captured on average each time. We took data on bm (accuracy = 0.1g; Tanita, digital balance M1479V, Japan), sex, reproductive state and age. Pregnant females were recognized by palpation of the abdomen, and lactating females by enlarged nipples surrounded by hairless skin. Juveniles were identified by the transparence of the cartilaginous plates in their metacarpal-phalangeal joints [50]. We extracted 50–100 μl of blood from the caudal vein in the interfemoral membrane of each bat following a standard method [51]. Low pressure was applied to the puncture site after extraction to prevent or stop bleeding. Blood samples were preserved in 70% ethanol and stored at room temperature until analysis [52]. Bats were released at their roosting sites after sampling. Capture and experiments were officially approved by the Environmental Council of the Junta de Andalucía (permit issue dates: December 12^th^ 2003, February 2^nd^ 2005). At the time we conducted this study, this was the only authority in charge of approving field research using animals in Andalusia, and no additional ethics approval was required. The latter was first imposed in Spain on February 1^st^, 2013 by the regulation “Real Decreto 53/2013”. The Ethics Committee on Animal Experimentation of the Doñana Biological Station (CEEA-EBD) was first created in 2013 to comply with this regulation.

Data on *Nyctalus lasiopterus* from Popa-Lisseanu et al. [37] used in this study were collected using the same capture methodology and blood sampling and preservation protocol as described above, although not with the same periodicity.

### Stable isotope analysis

We analyzed stable carbon and nitrogen isotope ratios of blood at the Stable Isotope Laboratory of the Estación Experimental del Zaidín (CSIC, Granada). Ethanol was removed from samples prior to analysis by freeze-drying. Samples were combusted at 1020ºC using continuous-flow system by means of an EA-IRMS elemental analyzer (Carlo Erba 1500NC) on line with a Delta Plus XL mass spectrometer, using helium as the carrier gas. The stable isotope composition was reported as δ values per mil (‰) using the formula: δ*X* = [(*R*_sample_—*R*_standard_)/*R*_standard_] ·1000; where *X* is either ^13^C or ^15^N, and *R* the proportion ^13^C /^12^C or ^15^N /^14^N ratios. The standard reference for carbon is PDB (Pee Dee Belemnite, a marine fossil) and for nitrogen (AIR) an average of ^15^N /^14^N from atmospheric air.

Commercial CO_2_ and N_2_ were used as working standards. We used two internal standards, EEZ-18 (shark cartilage), with δ^13^C of -13.96‰ and δ^15^N of +14.16‰, and UR-05 (urea), with δ^13^C of -43.82‰ and δ^15^N of -1.02‰. Internal laboratory standards are contrasted with the IAEA international references for carbon NBS-28, NBS-29, NBS-20 (carbonates) and NBS-22, IAEA-CH-7, IAEA-CH-6 (organic material), and for nitrogen IAEA-N-1, IAEA-N-2, NO-3, USGS32, USGS34 and USGS35. All samples were analysed by duplicate on different days. The overall precision of analyses was ± 0.1‰ for both δ^13^C and δ^15^N.

### Aridity index

The term aridity generally refers to the deficiency of available water in the ecosystem, whereby temperature and precipitation are two critical factors. There is however no consensus on the best way to define and measure aridity, and a large number of aridity indices have been proposed to date [53]. We developed our own monthly aridity index (AI) for our study area to explore potential relationships between environmental conditions and the monthly variation of δ^13^C and δ^15^N in bat blood. We calculated AI by dividing the monthly mean of daily maximum temperatures by the monthly precipitation plus 10 mm (to avoid division by 0 for the rainless summer months). Thus, the larger the value of AI for a particular month, the drier the climate in that month. We used maximum daily temperatures instead of daily means because the former are likely to be a better predictor of water stress and stomatal closure for plants in a climate with extreme hot summers such as the study area. We defined month as the period between day 15 of the previous month and day 14 of the actual month, since monthly blood sampling took place on day 15 (±2 days). We assumed a delayed response of bat blood isotope values to environmental conditions. Correlations between climatic seasonality and temporal variation in stable isotope values of plant material with time lags from 0 up to several months have been reported [20]. It may take insects as fast as one day up to several weeks to reflect changes in the isotopic values of their plant diets [54, 55]. Furthermore, bat blood has been shown to integrate the isotopic values of the diet consumed during the previous 1–3 months [56]. Similar to the approach conducted by other authors [57–60], we performed linear regression analyses between the AI and δ^13^C and δ^15^N of bat blood over a range of plausible time lags (0–5 months) by shifting each month’s AI back in time by 0 to 5 months, to find the time lag with the highest correlation.

Climatic data were obtained from the Doñana Biological Reserve [61].

### Statistical model

We created two general linear models (LM) in R.3.1.0 [62] to test which factors influenced monthly δ^13^C and δ^15^N values in bat blood (respectively the response variable in each model). We selected the following predictor variables for both models: month, species (sp; to test differences in species’ response, each species occupying a specific foraging niche), sex class (sex; to test the effect of reproduction), body mass (bm; indicator of fat deposition prior to hibernation), aridity index (AI), age class (age; juveniles, J, vs. adults, A), and the interactions between sex and age (sex*age) and between body mass and age (bm*age; since autumn increase in bm of juveniles is a result of growth in addition to fattening). Given that early stages of pregnancy cannot be identified through palpation, and pregnancy compromises the use of bm as an estimator of fat accumulation, we created the categorical variable “reproduction factor” (reproF) to filter out bm values of potentially pregnant females. It took the value “yes” (y) when a pregnancy was possible (for adult females of all species between March and June, and for all adult female *M. myotis* irrespective of month, since they can reproduce throughout the year in the studied roost), and “no” (n) when otherwise. The interaction between bm and reproF (bm*reproF) was thus incorporated in the model.

Before running the models we checked for independence of variables by calculating Pearson’s product moment correlation *r* between all single predictor variables. Predictor variables with |*r*| >0.75 are considered strongly correlating and should not be entered simultaneously. Given that month and AI strongly correlated, we used AI in all further analysis as a surrogate for month. None of the other variables showed strong correlations, so all variables except month were entered into the models as described above. Further, we used generalized additive models with three knots (GAM; package mgcv [63]) to visually check the linearity assumption of the variables so that non-linear variables could be turned into suitable parametric terms. All variables showed linear behaviour. We used the Kolmogorov-Smirnov normality test with Lilliefors correction to test for homogeneity in residuals of the final models (package nortest [64]).

For all analyses we set the significance level for the *P*-value at 0.05.

## Results

We obtained blood samples from 627 bats: 154 *Eptesicus isabellinus* (132 adult females, 8 adult males, 14 juveniles), 284 *Myotis myotis* (103 adult females, 161 adult males, 20 juveniles) and 189 *Miniopterus schreibersii* (79 adult females, 99 adult males, 11 juveniles). Additionally, we used blood isotopic data of 223 *Nyctalus lasiopterus* (176 adult females, 18 adult males, 29 juveniles) from the study by Popa-Lisseanu et al. [37]. *M. schreibersii* and *M. myotis* could be captured on emergence year-round (no hibernation) and data for these species could therefore be collected throughout the whole study period. No individuals of *E. isabellinus* and *N. lasiopterus* emerged from the roosts between November—February (hibernation period) and no data on these species could be obtained for this period.

**Fig 1:**
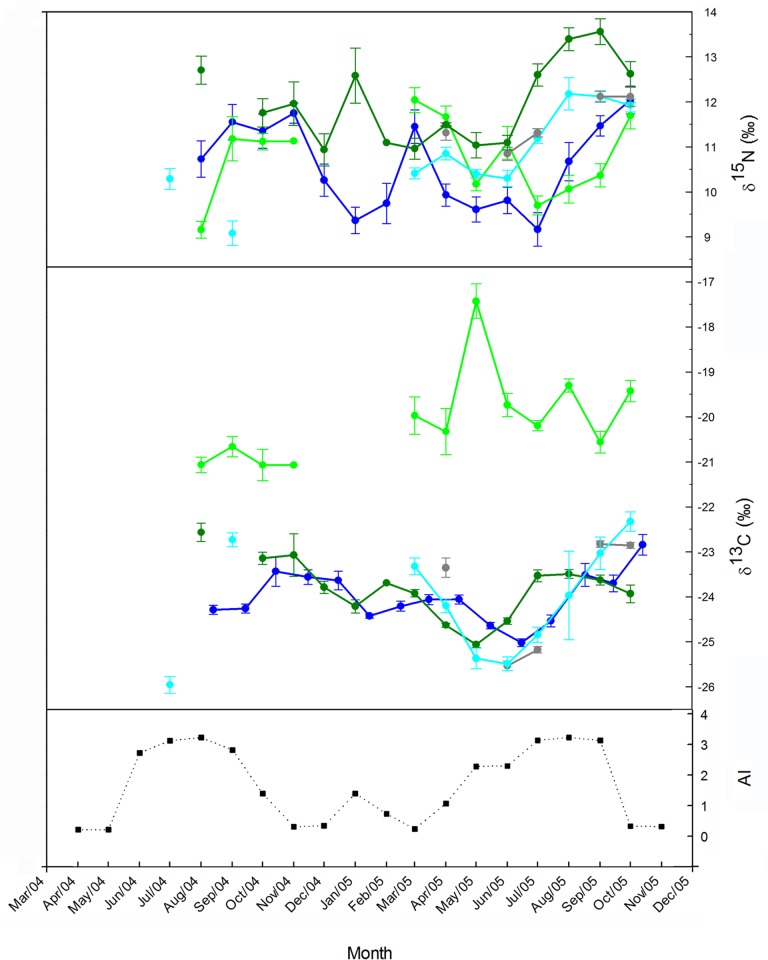
Monthly variation in AI, blood δ^13^C and δ^15^N (mean ± SE) of different bat species in southern Spain. Includes data from Popa-Lisseanu et al. [37]. Data of different years are plotted on the same scale for comparison. 2004–2005: *M. myotis* (blue), *M. schreibersii* (dark green), *E. serotinus* (light green); 2002–2003: *N. lasiopterus* (cyan); 2004: *N. lasiopterus* (grey).Monthly mean values are not joined when data of more than two months are missing.

For *M. myotis, M. schreibersii* and *N. lasiopterus*, monthly mean δ^13^C values of blood decreased from spring to summer by 1–2‰ and increased from the end of summer and continuously throughout autumn by 1.5–3‰. Monthly δ^13^C values of *E. isabellinus* did not conform to this pattern, but were 2–10‰ higher than for all other species and experienced the highest peak in May (Fig. 1). Therefore, we excluded *E. isabellinus* from the general model for δ^13^C, since its inclusion obscured the common pattern. Monthly mean δ^15^N values of all species including *E. isabellinus* increased throughout autumn by 2–3‰. An early-year drop (0.5–2‰) was also observed, but its timing differed between species (Fig. 1). We included all four species in the model for δ^15^N.

Most adult females were reproductive during the breeding period (e.g. 92% of *M. myotis* females in May, 75% of *E. isabellinus* females and 90% of *M. schreibersii* females in June). Pregnant or lactating female *M. myotis* were captured throughout most of the year (in November and continuously between January and July).

We found significant positive correlations between AI and both carbon and nitrogen stable isotope values of *M. myotis* and *M. schreibersii* (the two species for which we had winter data) after shifting the isotopic curves 1–4 months backwards to account for a time lag in the effect of climate on isotopic values. For *M. schreibersii*, significant correlations between AI and both δ^13^C and δ^15^N values were obtained at a time lag of two months (δ^13^C: *r* = 0.714, *p* = 0.00414; δ^15^N: *r* = 0.600, *p* = 0.0232), and for δ^15^N, also of 1 month (*r* = 0.587, *p* = 0.0272). For *M. myotis*, significant correlation between AI and δ^13^C were obtained at time lags of three months (*r* = 0.777, *p* <0.001), 2 months (*r* = 0.585, *p* = 0.0221) and 4 months (*r* = 0.531, *p* = 0.0221), and between AI and δ^15^N for time lags of 2 months (*r* = 0.769, *p* < 0.001) and 3 months (*r* = 0.607, *p* = 0.0164). As the models required assuming the same time lag for all species, and as a 2-month time lag worked well for both the small (*M. schreibersii*) and the large species (*M. myotis*), we incorporated the factor AI into the LM of δ^13^C and δ^15^N after accounting for the time lag of two months.

### Linear model

**Fig 2:**
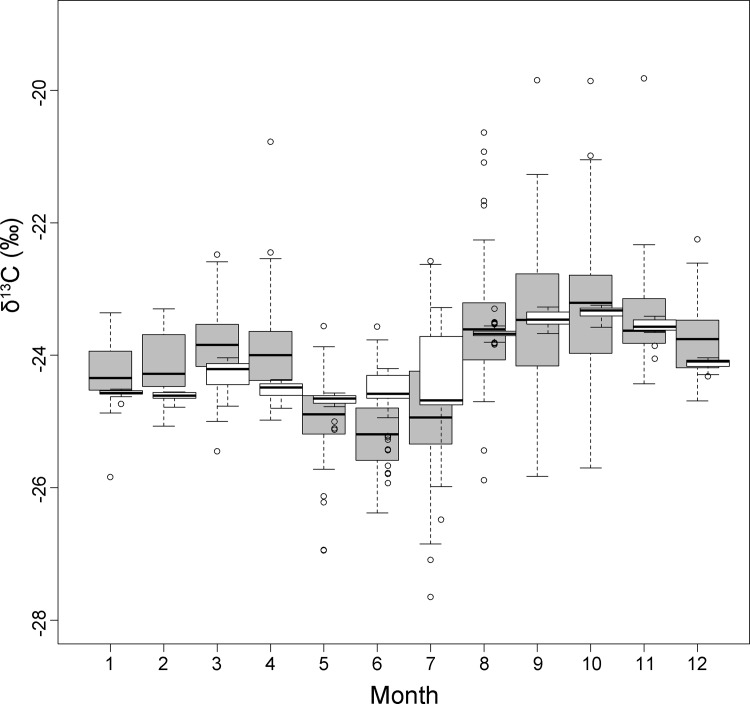
Box plots showing monthly median δ^13^C values of the overall model combining three bat species (*M. myotis, M. schreibersii* and *N. lasiopterus)*. Observed δ^13^C values: grey; predicted: white.

**Fig 3:**
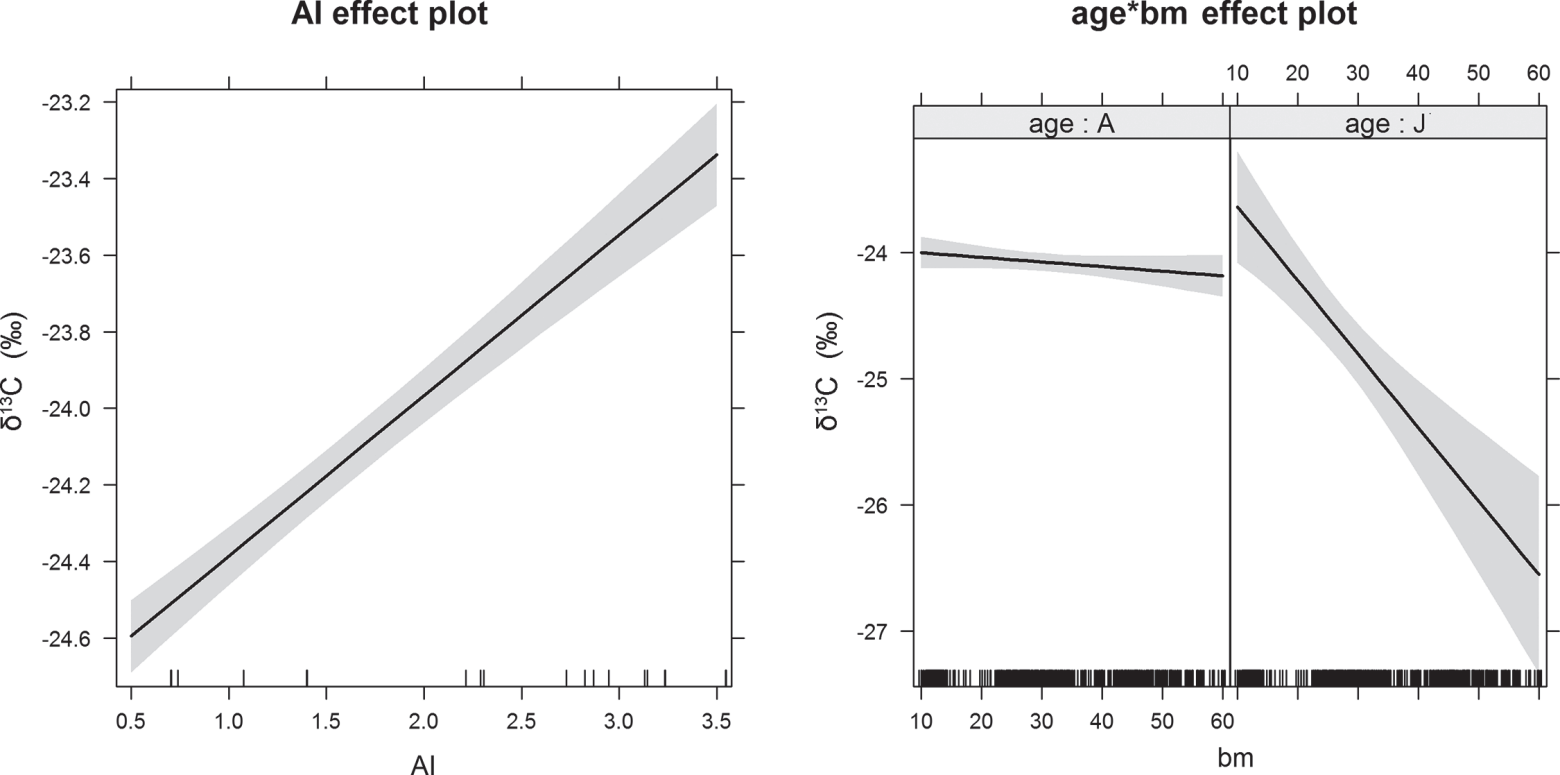
Effect plots in the linear model fit to the δ^13^C data for the two significant factors aridity index (AI) and the interaction between body mass and age (bm*age). A 95-percent pointwise confidence interval is drawn around the estimated effect. A: adults, J: juveniles.

The full model for δ^13^C was statistically significant (F = 31.81, df = 10 and 679, p < 0.0001) and accounted for ca. 30% of the variance in δ^13^C (adjusted R^2^ = 0.31) (Table 1). Monthly δ^13^C values predicted by the model and monthly observed δ^13^C values of bat blood (all species except *E. isabellinus*) are represented as a box and whisker plot in Fig. 2. Among the independent variables, the significant predictors of δ^13^C were AI (p < 0.0001), the interaction between bm and age (p < 0.0001), and age as single factor (p < 0.01) (Table 1, Fig. 3).

**Table 1:** Results of a linear model explaining the dependence of δ^13^C on the independent variables.

	Estimate	Std. Error	t value	Pr(>|t|)
(Intercept)	-24.466	0.340952	-71.758	< 2e-16 ***
spMsc (vs. spMmy)	-0.05887	0.190486	-0.309	0.75739
spNla (vs. spMmy)	0.148543	0.214644	0.692	0.48915
AI	0.434946	0.032724	13.292	< 2e-16 ***
reproFy (vs. reproFn)	-0.04384	0.268669	-0.163	0.87044
bm	-0.01152	0.010792	-1.067	0.28629
ageJ (vs. ageA)	0.996006	0.385531	2.583	0.00999 **
sexm (vs. sexf)	-0.03813	0.113652	-0.336	0.73735
reproFy:bm	0.004063	0.006628	0.613	0.54011
ageJ:sexm	-0.23178	0.257655	-0.9	0.36866
bm:ageJ	-0.05447	0.012416	-4.387	1.33e-05 ***

Residual standard error: 0.8892, d.f. = 679 (6 observations deleted due to missingness), multiple r^2^ = 0.3191, adjusted r^2^ = 0.309, F_10,679_ = 31.81, p < 0.0001. spMsc: *M. schreibersii*, spNla: *N. lasiopterus*, spMmy: *M. myotis*. AI: aridity index; reproFy (vs. reproFn): reproduction factor, “yes” vs. “no”; bm: body mass; ageJ (vs. ageA): age, juveniles vs. adults; sexm (vs. sexf): sex, males vs. females; reproFy:bm: interaction between reproduction factor (“yes” vs. “no”) and body mass; ageJ:sexm: interaction between age (juveniles vs. adults) and sex (males vs. females); bm:ageJ: interaction between bm and age (juveniles vs. adults).

*** *p* < 0.001

** *p* < 0.01.

The full model for δ^15^N was statistically significant (F = 31.11, df = 11 and 832, p < 0.0001) and accounted for ca. 28% of the variance in δ^15^N (adjusted R^2^ = 0.28) (Table 2). Monthly δ^15^N values predicted by the model and monthly observed δ^15^N values of bat blood (all species) are represented as a box and whisker plot in Fig. 3. Among the independent variables, the best predictor of δ^15^N was AI (p < 0.0001), followed by age (p = 0.01) and by the interaction between bm and age (p < 0.01). Additionally, there were significant differences between species (Table 2).

**Table 2:** Results of a linear model explaining the dependence of δ^15^N on the independent variables.

	Estimate	Std. Error	t value	Pr(>|t|)
(Intercept)	10.11304	0.402436	25.13	< 2e-16 ***
spMmy (vs. spEis)	0.271666	0.176169	1.542	0.12344
spMsc (vs. spEis)	1.483578	0.226016	6.564	9.21e-11 ***
spNla (vs. spEis)	1.857984	0.347835	5.342	1.19e-07 ***
AI	0.39494	0.048379	8.163	1.20e-15 ***
reproFy (vs. reproFn)	0.230018	0.349179	0.659	0.51025
bm	-0.02334	0.014674	-1.59	0.11216
ageJ (vs. ageA)	1.448559	0.479354	3.022	0.00259 **
sexm (vs. sexf)	0.228303	0.152351	1.499	0.13437
reproFy:bm	-0.0093	0.009258	-1.005	0.31533
ageJ:sexm	-0.20562	0.352256	-0.584	0.55956
bm:ageJ	-0.04715	0.016258	-2.9	0.00383 **

Residual standard error: 1.36, d.f. = 832 (6 observations deleted due to missingness), multiple r^2^ = 0.2914, adjusted r^2^ = 0.2821, F_11,832_ = 31.11, p < 0.0001. spMmy: *M. myotis*, spMsc: *M. schreibersii*, spNla: *N. lasiopterus*; spEis: *E.isabellinus*. AI: aridity index; reproFy (vs. reproFn): reproduction factor, “yes” vs. “no”; bm: body mass; ageJ (vs. ageA): age, juveniles vs. adults; sexm (vs. sexf): sex, males vs. females; reproFy:bm: interaction between reproduction factor (“yes” vs. “no”) and body mass; ageJ:sexm: interaction between age (juveniles vs. adults) and sex (males vs. females); bm:ageJ: interaction between bm and age (juveniles vs. adults).

*** *p* < 0.001

** *p* < 0.01.

## Discussion

Measurements on the relative abundance of naturally occurring stable isotopes (stable isotope analysis) have been used for over twenty years in terrestrial ecology as a means to trace wildlife diets, especially to monitor trophic shifts or changes in diet composition. However, little attention has been given to identifying sources of temporal and seasonal isotopic variation of consumers’ tissues in terrestrial ecosystems. We investigated sources of seasonal isotopic variation in terrestrial high-level consumers, three insectivorous bat species occupying different foraging niches and one seasonally insectivorous/carnivorous bat species.

**Fig 4:**
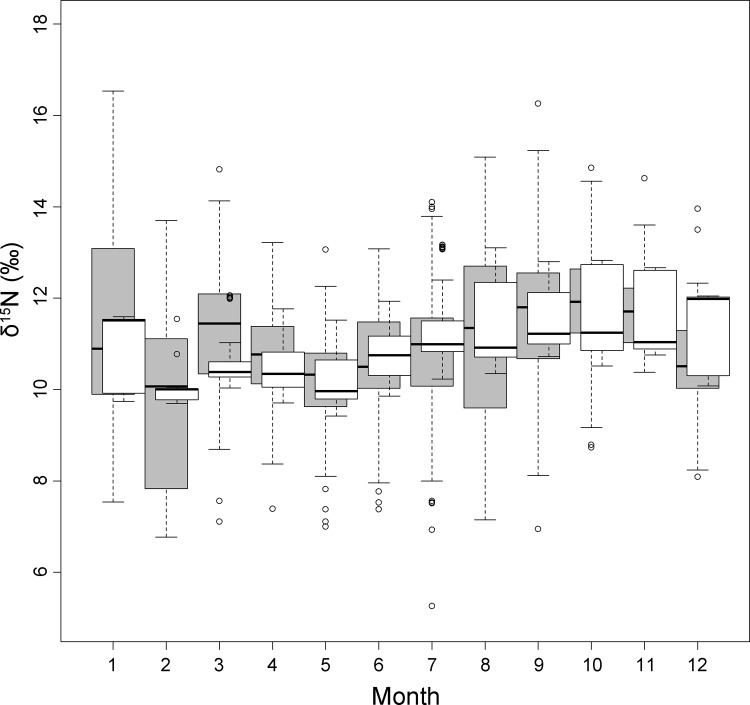
Box plots showing observed and predicted monthly median δ^15^N values of the overall model combining four bat species (*M. myotis, M. schreibersii, E. serotinus* and *N. lasiopterus)*. Observed δ^15^N values: grey; predicted: white.

The full linear models created to test the effect of species, reproduction, age, body mass changes related to hibernation, and climatic seasonality, were statistically significant and explained ca. 30% and ca. 28% of the variance in δ^13^C and δ^15^N values respectively. The overall models (combining all species for δ^15^N, and all but *E. isabellinus* for δ^13^C) supported the generality of the early spring and autumn enrichments in both isotopes (Figs. 1, 2, and 4). The existence of a common baseline despite strongly differing dietary habits of the species tested and despite the inclusion of different sampling years suggest a common, systematic source of isotopic variation in all bats. The overall isotopic change for each species was large enough to potentially be interpreted as a shift in trophic position, i.e. in the magnitude of one trophic step for δ^15^N (2.6‰ to 3.1‰) and many trophic steps for δ^13^C (2.2‰ to 3.6‰) considering the mean tissue-diet enrichment measured in Neotropical bats fed a protein-rich diet (3.3 ± 0.2‰ for δ^15^N and 0.1 ± 0.1‰ for δ^13^C) [56].

Only *E. isabellinus* δ^13^C values were an exception to the general pattern, as they were 2–10‰ higher than in the other species, with a peak of -17‰ in May (Fig. 1). These values indicate a food web partly derived from plants with a C4 photosynthetic pathway, which typically have δ^13^C around -14‰ [65], while the other bat species’ δ^13^C values are in the typical range of a C3-based food web (δ^13^C of C3 plants is around -27‰) [65]. The sampled *E. isabellinus* population roosts near extensive irrigated corn fields (a plant with C4 photosynthetic pathway) in Alcalá del Río [48], and may be foraging in this type of habitat. Even if *E. isabellinus* were subject to the same causes of seasonal variation in δ^13^C as the other species, the input of a diet based on a C4 food web would obscure their effect.

**Fig 5:**
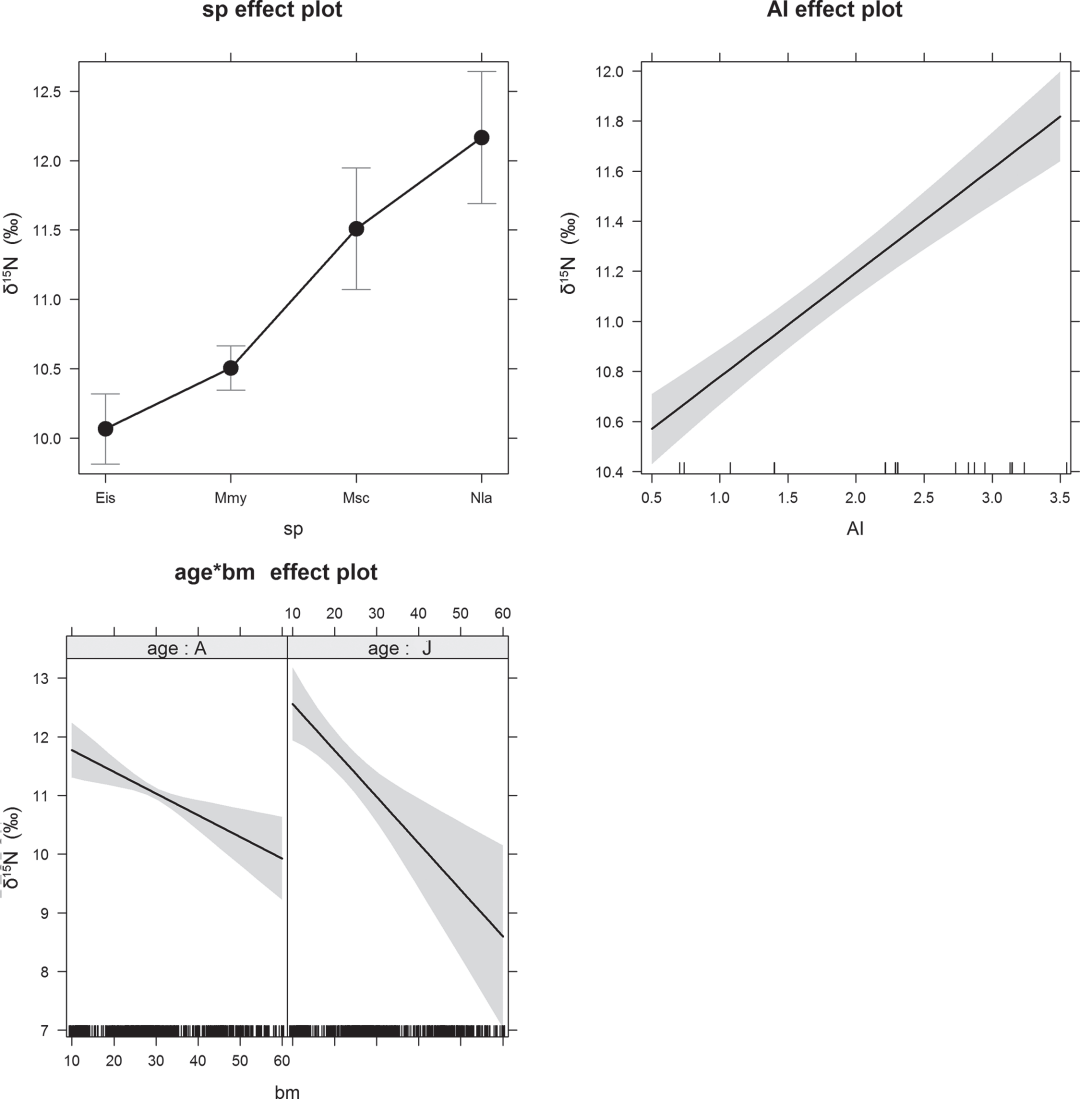
Effect plots in the linear model fit to the δ^15^N data for the three significant factors species (sp), aridity index (AI) and the interaction between body mass and age (bm*age). A 95-percent pointwise confidence interval is drawn around the estimated effect. A: adults, J: juveniles. Eis: *E. isabellinus*; Mmy: *M. myotis*; Msc: *M. schreibersii*; Nla: *N. lasiopterus*.

Although we did not collect simultaneous data on food consumption or foraging behavior, our results do not support seasonal changes in trophic position due to dietary shifts to be the cause of the observed seasonal isotopic patterns. Given the concurrence of the spring and autumn isotopic enrichments in all bat species, we can conclude that they are not driven in *N. lasiopterus* by seasonal carnivory, as previously believed [37]. Other concomitant changes in trophic position in all species are difficult to endorse, since each species occupies a different foraging niche: ground arthropods (*M. myotis*) vs. hard, medium—large aerial insects (*E. isabellinus*) vs. soft, small—medium aerial insects (*M. schreibersii*) vs. aerial insects and birds (*N. lasiopterus*). Despite this, species had no effect on the model for δ^13^C, and for δ^15^N, the species clustered in two groups that differed significantly from one another, with no significant difference between the two species within each group (Fig. 5). *N. lasiopterus* and *M. schreibersii* were enriched in ^15^N relative to *M. myotis* and *E. isabellinus*. While the ^15^N enrichment of *N. lasiopterus* can be explained by its carnivorous habits, *M. schreibersii* in our study area could be consuming large amounts of blood-feeding Culicidae (Culicidae are a component of the diet of the species [42]).

The models allowed us to test the effect of bat physiology on seasonal isotopic fluctuations. The physiology of temperate bats is marked by two main seasonal events: reproduction and hibernation. Pregnancy and lactation could affect isotopic values of females in two opposite ways. On the one hand, the increased energy demands of gestating and lactating females could lead to nutritional stress, resulting in catabolism of body protein and ^15^N enrichment. There is some evidence of this effect in pregnant humans [66] and in lactating free ranging mammals [35, 67, 68]. On the other hand, some studies report a decrease in either only δ^15^N, only δ^13^C, or both δ^13^C and δ^15^N values of pregnant [31] or lactating females [30, 69]. This could be a consequence of the isotopic discrimination between mother’s milk and offspring during nutrient transfer, or, if depletion in ^15^N occurs, an effect of the net anabolic state associated with protein synthesis [31]. In this study, almost all female bats sampled were pregnant or lactating in May-June. If pregnancy and lactation affect isotopic values in bats, we would expect sex to have a significant effect on the model. However, this was not the case. Likewise, the models did not single out the species *M. myotis*, although it was the only one to experience winter reproduction. Our results therefore do not support physiological changes associated with reproduction to cause seasonal isotopic variation in bats.

Temperate bats usually retreat in winter to cold hibernacula and enter an energy-saving deep torpor, during which they consume fat stocked up before hibernation. Pre-hibernation fattening is evidenced by a steep increase in body mass during autumn [70]. Tissues of animals in hibernation, just like those of fasting and nutritionally stressed individuals, typically become enriched in ^15^N [33–35, 71, 72]. The most likely cause is the loss of ^14^N to urea, which, in the absence of nitrogen inputs from food, results in a higher proportion of ^15^N in plasma amino acids used for protein synthesis [73]. According to these observations, we could expect bat δ^15^N values to peak in late winter—early spring at the end of hibernation and to decrease progressively thereafter, maintaining low levels throughout summer and autumn, a period of increased food consumption. The changes in δ^15^N we observed were contrary to this expectation. Cherel et al. [72] reported a decrease in δ^13^C values in plasma of fasting penguins, which the authors speculated to be the result of an increase in the concentration of circulating lipids. Lipid content in blood of bats has been shown to increase as bats build up fat reserves before hibernation and to peak during winter dormancy [74], and lipids are relatively depleted in ^13^C [75]. We could thus predict δ^13^C values of bats to start declining during fat mobilization prior to hibernation, a pattern contrary to the observed. Most importantly, body mass had no effect on the models, despite high interindividual and interspecific variability in body mass changes. For example, by October female *E. isabellinus* in our study gained about 30% of their August bm, while bm of *M. myotis* and *M. schreibersii* did not increase throughout autumn; female *M. schreibersii* sampled in 2005 even lost about 9.5% of their August bm by October. Isotopic variation was thus independent of pre-hibernation fattening. Lastly, despite *M. myotis* and *M. schreibersii* skipping hibernation at the studied roost, these two species were not grouped together by the model explaining δ^15^N values, and species had no effect on the model explaining δ^13^C. Overall, our results do not support physiological changes related to hibernation to drive seasonal isotopic variation in bats.

The factor age, and the interaction between age and body mass, had an effect on both models. Juvenile bats were enriched by ca. 1‰ and 1.5‰ relative to adults for δ^13^C and δ^15^N respectively; the difference declined with increasing body mass of juveniles (Figs. 3 and 5). These results are consistent with previous studies that show isotopic enrichment in nursing offspring, probably resulting from isotope discrimination between mother’s milk and offspring, and a decline towards adult values as weaning progresses [71, 76, 77].

There was a striking correlation between stable isotope values in bat blood and seasonal climatic variation. The two annual peaks in AI, a low peak in January and a broad, high peak at the end of summer, where followed ca. 2 months later by corresponding peaks in blood δ^13^C and δ^15^N values (Fig. 1). Accordingly, AI was the factor that best explained the models (Tables 1 and 2). Both δ^13^C and δ^15^N increased with increasing values of AI.

The most parsimonious explanation for the relationship between δ^13^C and AI is that bats reflect seasonal cycles in plant δ^13^C values, widely documented to correlate with water stress [11, 12, 14, 16]. Propagation of autotroph isotopic signals (both δ^13^C and δ^15^N) has been demonstrated in aquatic consumers at different trophic levels [8, 27, 28], and the same process is assumed to occur in terrestrial ecosystems [8], although not yet reported to our knowledge. In a similar habitat in ca. 250 km distance to our study area, a Mediterranean oak woodland in Portugal, seasonal changes in δ^13^C of ecosystem respiration (δ^13^C_R_, which typically correlates with leaf δ^13^C) resembled in temporality, direction and magnitude the observed δ^13^C fluctuations of bat blood [59]. Model results for δ^13^C thus strongly support that climatic seasonality, mediated through concomitant oscillations in plant isotopic values, drive seasonal isotopic variation in bats.

The same propagation effects, from primary producers up to bats, could explain δ^15^N cycles in bat blood. In fact, δ^15^N values in terrestrial herbivores have also been shown to correlate negatively with rainfall over geographical climatic gradients. This pattern was previously thought to be the result of a physiological response of animals to water stress in arid regions, e.g. improved water recycling through protein catabolism inducing enrichment in ^15^N [36], a hypothesis that could also explain correlation between aridity and δ^15^N of bats in our study area. However, there is growing support that the relationship between herbivore δ^15^N values and geographical climatic gradients is not an effect of physiology, but merely the reflection of the relationship between environmental conditions and plant diet [78, 79]. Although the correlation between plant δ^15^N and aridity is well documented over geographical scales, it is unclear how this pattern works at short temporal scales [80]. This is partly because the mechanisms by which environmental variables affect N isotope discrimination in plants are not well understood [17]. Correlations between plant δ^15^N and seasonal changes in temperature and/or rainfall have been repeatedly reported in recent years [23–25, 81], but the directionality is not consistent between studies. Soil and plant δ^15^N dynamics are further complicated by agricultural practices [25]. Although our results suggest that aridity-induced enrichment in plant and consumer δ^15^N values occurs not only over geographical, but also over short temporal (seasonal) scales, we lack a mechanistic explanation for this phenomenon.

Our main interpretation that bat isotope values reflect environmental baselines implies that insects constituting the bats’ diet consume fresh plant material that incorporates the current aridity-induced changes in δ^13^C and δ^15^N throughout the year. Mature leaves are unlikely to reflect environmental changes in their δ^13^C values; however, phloem sap δ^13^C in Mediterranean tree species has been shown to correlate with environmental parameters even in late summer when leaves are mature [82]. Additionally, flowering or fruiting plant species and their associated insect visitors can be found year-round in Mediterranean shrubland and herbaceous plant communities [83, 84]. This means that changes in aridity are likely to be reflected in producer and consumer isotope values not only during spring leaf emergence, but also throughout summer and autumn.

An alternative hypothesis to explain δ^15^N patterns in bat blood is that bats in our study area, independent of species, simultaneously shift to the same types of foraging habitat seasonally. For example, dry conditions may force foraging bats to move from natural landscapes to cultivated fields or to aquatic habitats, often with higher δ^15^N values [85], to follow peaks in prey density. Consistent with this hypothesis, *N. lasiopterus* (belonging to the same population that provided data for this study) were shown to forage in riparian and partly cultivated marshland habitats during summer and autumn, more than doubling their home ranges relative to spring [86]. This hypothesis however does not explain the coincidence between δ^13^C trends and δ^15^N trends.

Despite the difficulty in satisfactorily explaining δ^15^N dynamics with the currently available knowledge, our study is the first to demonstrate systematic seasonal isotopic fluctuations in terrestrial consumers that can be related to environmental variation. We corroborate the conclusion of Woodland et al. [8] that temporally dynamic isotopic baselines should be investigated prior to the application of stable isotopes in ecology, to avoid biased conclusions on trophic relationships. Stable isotope analysis is currently used to provide answers to a wide range of questions on diet and foraging strategies [87–89], migration [90–92], physiology [93], host-parasite interactions [94], wildlife management [95] and conservation of threatened species [96], among others. To improve the predictive precision of this technology, further research on how consumers integrate changes in ecosystem isotopic values over different time scales is needed.
